# The chemokine scavenging receptor D6/ACKR2 is a target of miR-146a in thyroid cancer

**DOI:** 10.18632/genesandcancer.141

**Published:** 2017-05

**Authors:** Francesco Pacifico, Alessio Lepore, Stefano Mellone, Luca Sanguigno, Giorgia Federico, Adelaide Greco, Arturo Brunetti, Antonio Leonardi

**Affiliations:** ^1^ Istituto di Endocrinologia e Oncologia Sperimentale, CNR, Naples, Italy; ^2^ Dipartimento di Medicina Molecolare e Biotecnologie Mediche, Federico II University, Naples, Italy; ^3^ Dipartimento di Scienze Biomediche Avanzate, Federico II University, Naples, Italy; ^4^ CEINGE Biotecnologie Avanzate, scarl, Naples, Italy; ^5^ Istituto di Biostrutture e Bioimmagini, CNR, Naples, Italy

**Keywords:** D6/ACKR2 receptor, chemokines, miR-146a, NF-κB, thyroid cancer

## Abstract

We have previously shown that miR-146a, a NF-κB-regulated microRNA, is strongly expressed in human specimens and cell lines derived from anaplastic thyroid carcinomas (ATC) where it mediates some of the NF-κB pro-tumorigenic functions. By using a bioinformatic analysis, we identified the chemokine scavenger receptor D6/ ACKR2 as a target of miR146a in human ATC. We found that the expression of D6/ ACKR2 was up-regulated in miR-146a-null ATC cell lines and that the 3’ UTR of D6/ ACKR2 mRNA was able to inhibit its expression in parental, but not in miR-146a-null ATC cells. Since human specimens from primary ATC showed a low expression of D6/ ACKR2 compared to normal thyroid tissues, we analyzed the effects of D6/ACKR2 over-expression in ATC cells. Different chemokines added to the conditioned medium of D6/ACKR2 over-expressing ATC cells partially failed to drive *in vitro* monocyte migration, and tumors derived from the injection of the same cells in nude mice showed a decreased number of infiltrating macrophages.

Taken together, these results indicate that ATC cells down-regulate D6/ACKR2 expression through miR-146a activity to sustain leukocyte trafficking inside tumor microenvironment and shed light on a novel mechanism by which NF-κB indirectly inhibits the expression and the function of anti-tumorigenic gene in thyroid cancer.

## INTRODUCTION

The tumor microenvironment plays a pivotal role in the establishment and further development of cancer. It is composed of a number of different cell types including stromal fibroblasts, endothelial cells and immune cells which synergistically with neoplastic cells deeply contribute to tumor growth and to the multiple stages of tumor progression [1]. Infiltrating immune cells represent one of the most abundant and important components of tumor microenvironment so much so that almost all adult solid tumors contain infiltrates of diverse leukocyte subsets including both myeloid- and lymphoid-lineage cells [2], whose complexity and activation status vary depending on the tissue localization and stage of malignancy [3, 4]. Tumor infiltrating immune cells supply direct and indirect mitogenic growth mediators that stimulate proliferation of neoplastic cells, as well as other stromal cell types in their proximity [5], and express diverse classes of proteolytic enzymes that can modify the structure and function of extracellular matrix thus allowing tumor invasion and metastasis [6].

The presence of immune cells, other than stromal fibroblasts and endothelial cells, strongly indicates that the inflammatory response plays a strategic role in the onset of cancer. After all, epidemiological studies have well established that chronic inflammation predisposes to different forms of cancer, thereby supporting the intimate relationship between inflammation and cancer. In particular, the interplay between epithelial and inflammatory cells is thought to be crucial for the genesis and the establishment of carcinomas. One of the main actors in inflammatory process is NF-κB which, by regulating the expression of different cytokines and chemokines in inflammatory cells, stimulates the growth and its own activity in epithelial cells. Thus, NF-κB establishes a network that, after prolonged time, can lead epithelial cells to undergo malignant transformation [7]. Once activated in neoplastic cells, NF-κB promotes transcription of a number of genes that on one hand contribute to the establishment of its constitutive activation by an autocrine fashion, and on the other hand allow the recruitment of inflammatory and immune cells by a paracrine fashion, thereby facilitating the activity of tumor neighbouring cells [8].

D6, also called chemokine binding protein 2 (CCBP2) or atypical chemokine receptor 2 (ACKR2), is a constitutively internalizing receptor able to bind and to scavenge almost all inflammatory CC chemokines [9, 10]. It is expressed by lymphatic endothelial cell [11], by trophoblasts in the placenta [12] and by some leukocytes such as alveolar macrophages [13] and innate-like B cells [14], while its expression in neoplastic tissues inversely correlates with tumor aggressiveness [15, 16]. Moreover, D6/ACKR2-deficient mice have increased susceptibility to cutaneous tumor development [17] and showed increased susceptibility to colitis-associated cancer [18]. D6/ACKR2 knockout mice have increased number of circulating inflammatory monocytes [19] and defects in lymphatic vessel density and function [20] compared to wild type mice, and also exhibit exacerbated inflammatory reactions in barrier tissues very likely because of the lack of chemokine clearance, increased infiltration of inflammatory cells and lack of inflammation resolution.

We have demonstrated that miR-146a expression is up-regulated in human anaplastic thyroid carcinomas in a manner that parallels the NF-κB basal activity in the same tumors. The inhibition of miR-146a in anaplastic thyroid carcinoma cells determined the increase of their susceptibility to chemotherapeutic drugs-induced apoptosis and the lost of their oncogenic potential [21]. Therefore, we focused our interest on the molecular mechanisms by which miR146a played its role in thyroid cancer. We identified D6/ACKR2 as a target of miR-146a repressive activity in anaplastic thyroid carcinoma cells and we found that the receptor was expressed in neoplastic thyroid specimens at much less extent than in normal thyroid tissue. The exogenous expression of D6/ACKR2 receptor in anaplastic thyroid carcinoma cells determined a partial lost of their chemo-attractant properties versus monocytes and macrophages.

## RESULTS

### Identification and characterization of D6/ACKR2 as a target of miR-146a

**Figure 1:**
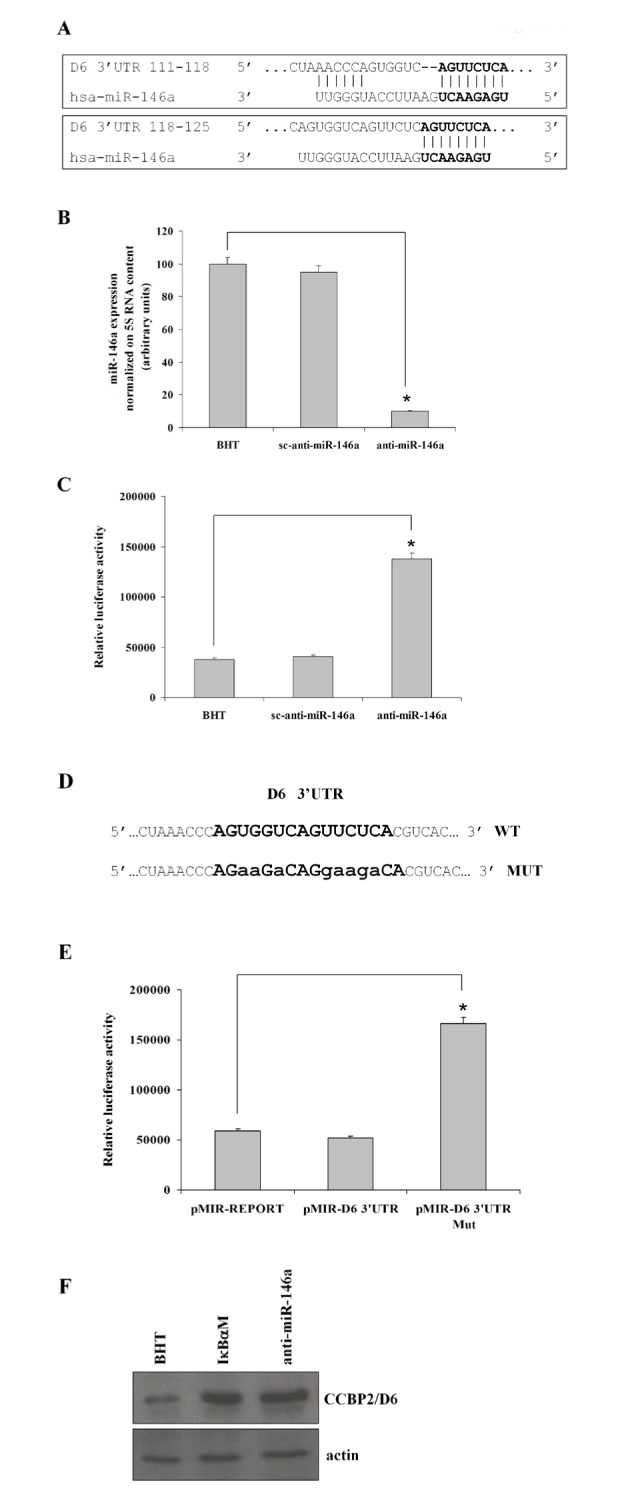
Identifcation and validation of D6/ACKR2 as a target of miR-146a in human ATC cells TargetScan 7.1 analysis showed that the 3'UTR of D6/ACKR2 mRNA contained two adjacent sites between nucleotides 111-125 for miR-146a binding (A, bolded sequences). B) miR-146a mRNA levels in parental, scrambled and anti-miR146a transfected BHT101 cells. The error bars represent technical replicates within a single experiment. Each experiment has been repeated at least three times *, p<0,00001. C) The pMIR-D6 3'UTR plasmid, containing the 3'UTRs of D6/ACKR2 mRNA downstream to the luciferase gene was transfected in the indicated BHT101 cell lines for reporter assays. In addition, luciferase activity (E) was also analyzed on BHT101 cells transfected with pMIR-D6 3'UTR Mut, a plasmid similar to pMIR-D6 3'UTR containing a mutated form of the 3'UTRs of D6/ACKR2 mRNA (D, bolded lower sequence). A pMIR-REPORT-β-galactosidase vector was used to normalize for transfection efficiencies. The error bars represent technical replicates within a single experiment. Each experiment has been repeated at least three times. *, p<0,001. F) Endogenous D6/ACKR2 receptor up-regulation upon miR-146a silencing was confirmed by Western blot analysis on parental and anti-miR-146a BHT cells and on BHT IκBαM as a control (upper panel). Protein expression was normalized on actin content (lower panel).

To identify the potential targets of miR-146a in human ATC cells, we analyzed the TargetScan 7.1 database and found that the chemokine scavenging receptor D6/ACKR2 was a predicted target of miR-146a given that two adjacent sites for its binding were located between nucleotides 111-125 of D6/ACKR2 mRNA 3'UTR (Figure 1A). To validate D6/ACKR2 as a target of the miR-146a, we cloned the 3'UTR of D6/ACKR2 mRNA downstream to the luciferase gene in the pMIR-REPORT vector. The resulting plasmid pMIR-D6 3'UTR was transfected in the BHT101 cells and the luciferase activity was measured in the presence of the anti-miR146a or in the presence of a scrambled anti-miR. As shown in figure 1B and C, BHT101 cells transfected with pMIR-D6 3'UTR showed very low levels of luciferase activity that was restored in the presence of the anti-miR-146a. To further confirm that the 3'UTR of D6/ACKR2 mRNA was targeted by miR-146a, we mutated the D6/ACKR2 3'UTR sequence recognised by the miR146a (Figure 1D). As shown in figure 1E, luciferase activity was low in BHT101 cells transfected with control plasmid or with pMIR-D6 3'UTR, but it strongly increased in pMIR-D6 3'UTR Mut transfected cells. Finally, we analyzed endogenous D6/ACKR2 expression by Western blot on cell lysates from parental and BHT-miR-146a-null cells. As shown in figure 1F, blocking the expression of miR-146a in BHT101 cells determined an increase of endogenous D6/ACKR2 protein levels, very similar to that observed in the same cells infected with a lentivirus carrying a cDNA encoding for a super-repressor form of IκBα that prevents NF-κB activation, thereby determining miR-146a down-regulation [21].

These results very likely indicate that D6/ACKR2 receptor is a target of miR-146a activity in anaplastic thyroid carcinoma cells.

### D6/ACKR2 expression in primary human thyroid carcinomas

**Figure 2:**
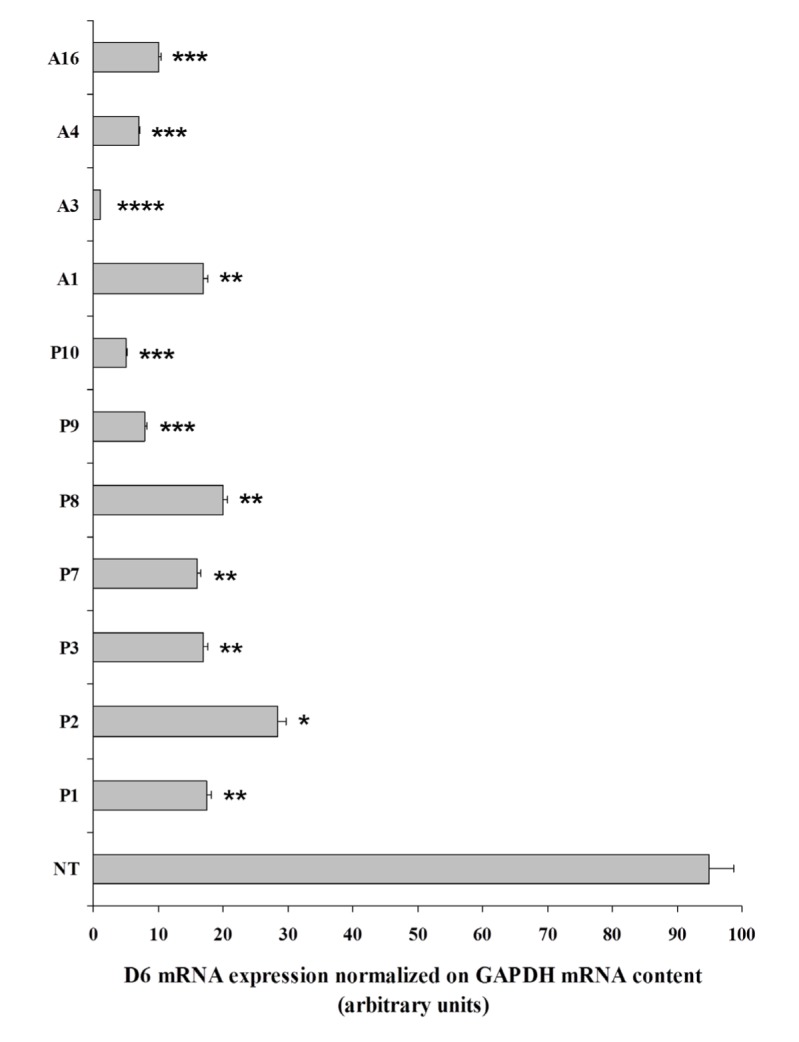
D6/ACKR2 receptor expression in primary human specimens from thyroid carcinomas Quantitative RT-PCR assays were performed to analyze D6/ACKR2 receptor mRNA expression in normal human thyroid (NT), papillary (P1, P2, P3, P7, P8, P9, P10) and anaplastic (A1, A3, A4, A16) thyroid carcinomas. D6/ACKR2 receptor mRNA expression was normalized on GAPDH mRNA content. The error bars represent technical replicates within a single experiment. Each experiment has been repeated at least three times Statistical analysis was performed by comparison between normal thyroid and tumors D6/ACKR2 mRNA content. *, p<0,001; **, p<0,0001; ***, p<0,00001; ****, p<0,000001

In a previous paper we demonstrated that miR-146a was strongly expressed in human tissue specimens from anaplastic thyroid carcinomas and almost undetectable in the normal counterpart [21]. Therefore, we decided to analyze D6/ACKR2 expression in normal thyroid tissue and anaplastic tumors, that represent the most undifferentiated histotype among thyroid neoplasms, and to extend our investigation also to human specimens from papillary thyroid carcinomas that, on the contrary, belong to the group of differentiated thyroid tumors. The qRT-PCR analysis showed that both papillary (P1, P2, P3, P7, P8, P9, P10) and anaplastic (A1, A3, A4, A16) carcinomas expressed lower levels of D6/ACKR2 receptor than those of normal tissue (NT), even though at different extent (Figure 2). In particular, the majority of papillary thyroid carcinomas displayed a 3- to 5-fold decrease of D6/ACKR2 mRNA, while three out of four anaplastic thyroid carcinomas exhibited a 10- to 50-fold decrease of D6/ACKR2 expression (Figure 2).

These data evidence the inverse correlation between the expression of miR-146a [21] and D6/ACKR2 receptor (Figure 2) in normal and neoplastic thyroids, confirming that D6/ACKR2 was down-regulated by miR-146a also in human specimens, and suggest that D6/ACKR2 expression is lost along with the de-differentiated state of thyroid carcinomas.

### Role of D6/ACKR2 receptor in anaplastic thyroid carcinoma cells

**Figure 3:**
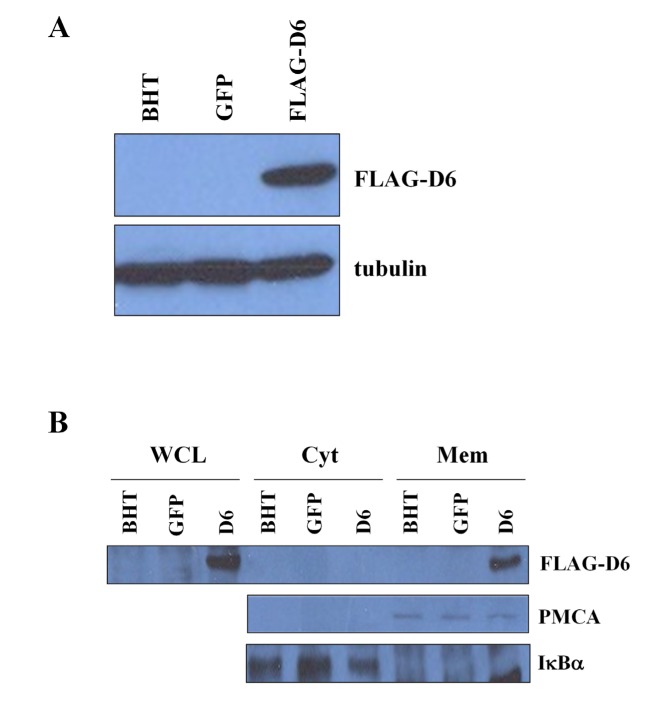
Exogenous D6/ACKR2 receptor localizes on the surface of BHT cells A) To over-express D6/ACKR2 receptor, BHT cells were infected with pLenti-CMV-GFP-2A-Puro lentiviral vector alone (BHT GFP) or containing the cDNA coding for human D6/ACKR2 fused in frame with FLAG tag (BHT FLAG-D6) (upper panel). Protein expression was analyzed by anti-FLAG antibody and normalized on tubulin content (lower panel). B) Cell fractionation of different BHT cell lines. Whole cell lysate (WCL), cytosolic (Cyt) and microsomal (Mic) fractions were analyzed by Western blot to dectect D6/ACKR2 localization by anti-FLAG antibody (upper panel). The purity grade of cell fractionation was assessed by the specific expression of two markers, the Plasma Membrane Calcium ATPase (PMCA) for the microsomal fraction (middle panel) and IκBα for the cytosolic fraction (lower panel).

To study the role of D6/ACKR2 in thyroid cancer, we infected the BHT101 cells with a lentivirus carrying the human D6/ACKR2 cDNA fused in frame with a FLAG sequence. As shown in Figure 3A, Western blot analysis of infected BHT101 cells (FLAG-D6) showed the expression of exogenous D6/ACKR2 that was absent in uninfected (BHT) and vector alone (BHT GFP) infected cells. To ensure that infected D6/ACKR2 was localized on the plasma membrane, we performed a cell fractionation of different BHT101 cell lines and analyzed by Western blot the expression of D6/ACKR2 in the cytosolic and microsomal fractions. Infected D6/ACKR2 was present in the microsomal fraction, that contained membrane vesicles from endosomes, plasma-membrane, Golgi, endoplasmic reticulum [22], whereas it was absent in the cytosolic one (Figure 3B, upper panel). The purity grade of cell fractionation was assessed by the specific expression of two markers, the Plasma Membrane Calcium ATPase (PMCA) for the microsomal fraction (Figure 3B, middle panel) and Iκ for the cytosolic fraction (Figure 3B, lower panel).

**Figure 4:**
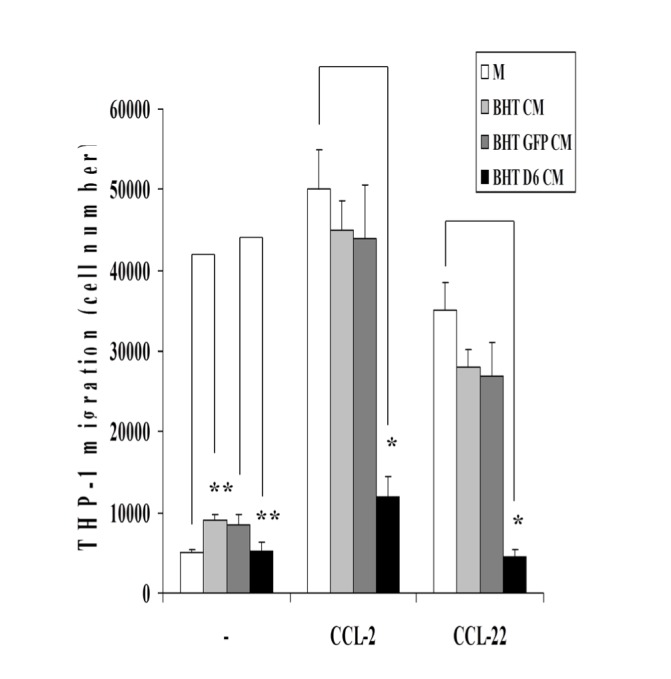
D6/ACKR2 receptor scavenging activity in infected BHT cells Conditioned media (CM) from BHT (light gray bars), BHT GFP (dark gray bars) and BHT D6 (black bars) cell lines challenged with 1.2 nM CCL-2 or CCL-22 and with no chemokines (-) were added to the lower compartment of a Boyden chamber to drive THP-1 monocytes migration from the upper compartment in a transwell assay. Medium only (M, white bars) in the absence or in the presence of 1.2 nM CCl-2 or CCL-22 was used as a control. The number of migrated THP-1 cells were counted in a Neubauer chamber. The error bars represent technical replicates within a single experiment. Each experiment has been repeated at least three times *, p<0,001 * *, p<0,01

Thus, to verify if BHT D6 cells expressed the functional receptor, we investigated its ability to scavenge chemokines from extracellular medium. To this end, conditioned media from BHT, BHT GFP and BHT D6 cell lines challenged with 1.2 nM CCL-2 or CCL-22 and with no chemokines (-) were added to the lower compartment of a Boyden chamber to drive THP-1 monocytes migration from the upper compartment in a transwell assay. Medium only in the absence or in the presence of 1.2 nM CCl-2 or CCL-22 was used as a control. Both CCL-2 and CCL-22 in the medium only as well in the conditioned media from all cell lines were able to attract THP-1 cells to the lower chamber, but at different extent (Figure 4). In fact, the number of THP-1 cells recruited upon challenging with CCL-2 and CCL-22-containing conditioned media from BHT D6 cells (Figure 4, black bars) decreased of ~70% compared to that driven by BHT (Figure 4, light grey bars) and BHT GFP (Figure 4, dark grey bars) conditioned media. In addition, Figure 4 also shows that the contribute of BHT and BHT GFP cells endogenous D6/ACKR2 in chemokine scavenging is poor given that the number of THP-1 migrated cells driven by BHT and BHT GFP cells conditioned media is only ~10% lower than that driven by chemokines in the medium only (compare white bars vs. light and dark grey bars). In the absence of CCL-2 or CCL-22 (Figure 4), THP-1 chemotaxis induced by BHT and BHT GFP cells conditioned media is increased respect to that induced by medium only (light and dark grey bars vs. white bars), but at much lower extent than exogenous CCL-2 or CCL-22-driven migration. D6/ACKR2 over-expression also decreases endogenous chemokine-driven THP-1 chemotaxis to the levels of control (Figure 4, - black bars vs. white bars). These findings suggest that the presence of the ectopic D6/ACKR2 receptor on the plasmamembrane of infected BHT101 cells determined the scavenging of both chemokines from extracellular medium and that the low basal expression of the receptor in BHT101 cells cannot account for a sustained chemokine internalization.

**Figure 5:**
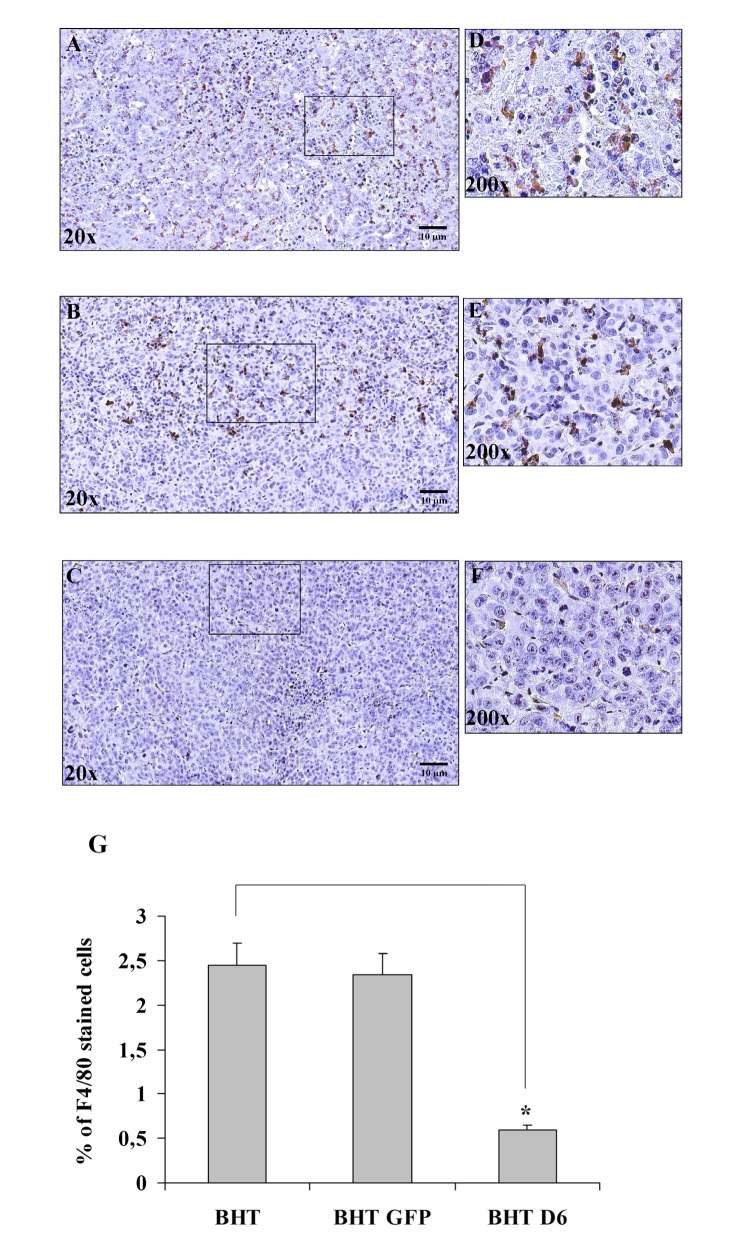
Immunohistochemistry of tumor xenografts from D6/ACKR2 deficient and proficient BHT cells Tumor xenografts isolated from mice injected with BHT (A, D), BHT GFP (B, E) and BHT D6 (C, F) stained with rat anti-mouse F4/80. A, B and C panels (20x magnification) represent panning shots of tumor specimens, while D, E and F panels (200x magnification) are enlargements of boxed areas of A, B and C panels. F4/80 positive macrophages are detected as brown stained cells on a background of blue unstained tumor cells. Results are ±SD of the number of positive F4/80 stained cells from six mice xenografts for each experimental group analyzed by ImageJ software (NIH, Bethesda MD, USA) (G).*, p<0,001

On the basis of these observations, we studied *in vivo* the role of D6/ACKR2 restoration in anaplastic thyroid carcinoma cells. BHT, BHT GFP and BHT D6/ACKR2 cells were injected subcutaneously in nude mice to induce tumor development and, after 3 weeks, the macrophages infiltrate was analyzed in tumor xenografts to evaluate if the presence of D6/ACKR2 on the surface of neoplastic cells could limit the number of macrophages in tumor microenvironment. The immunohistochemical analysis with an antibody that specifically targets the F4/80 marker of murine macrophages evidenced a remarkable macrophage infiltrate in tumor xenografts arised from BHT and BHT GFP cell injection (Figure 5A/D-B/E and Figure 5G), which strongly diminished in BHT D6-derived tumors (Figure 5C/F and Figure 5G ).

All together these findings indicate that D6/ACKR2 is a target of the miR146a and that restoring its expression in thyroid cancer cell lines decreased macrophage chemotaxis both *in vitro* and *in vivo*.

## DISCUSSION

In this paper we demonstrate that D6/ACKR2 receptor is a novel target of the NF-κB-regulated miR-146a in thyroid cancer. A number of evidences support this discovery: i) anaplastic thyroid carcinoma cell lines expressing high levels of miR-146a display low D6/ACKR2 expression and blocking miR-146a in the same cells restored D6/ACKR2 expression; ii) reporter assays prove that D6/ACKR2 mRNA 3'UTR contains a specific sequence targeted by miR-146a in anaplastic thyroid carcinoma cell lines; iii) human specimens from normal and neoplastic thyroid tissues show an inverse correlation between miR-146a expression and D6/ACKR2 content. Down-regulation of D6/ACKR2 expression by miR-146a warrants to thyroid cancer cells the property to recruit inflammatory cells *in vitro* and *in vivo*. In fact, conditioned media from BHT cells over-expressing D6/ACKR2 partially lose the ability to induce CCL-2- or CCL-22-driven monocytes migration in a transwell assays. Moreover, tumor xenografts from parental or control BHT cells show a strong macrophage infiltrate that considerably decreases in tumor microenvironment of BHT D6 cells-derived specimens. Thus, thyroid carcinomas maintain low D6/ACKR2 levels to sustain chemokine trafficking inside tumor microenvironment and to ensure tumor progression driven by the pro-oncogenic activity of recruited inflammatory cells.

The anti-oncogenic properties of D6/ACKR2 receptor have been reported in several types of human tumors. Its expression, in fact, correlates with a better outcome of gastric cancer [23] and cervical squamous cell cancer [24], inhibits lung cancer cells proliferation and tumorigenesis [25] and not only is down-regulated during breast cancer progression, but is also prognostic of a favourable disease-free survival rate in breast cancer patients [26]. In addition, in Kaposi sarcomas D6/ACKR2 delays tumor progression through inhibition of inflammatory chemokines CCL-2, CCL-5 and CCL-3 and reduces macrophage infiltration and angiogenesis [15]. As a consequence, in patients with high aggressive Kaposi sarcomas D6/ACKR2 is down-regulated [15]. The low levels of D6/ACKR2 in primary human thyroid carcinomas and its ability to decrease leukocytes recruitment *in vitro* and *in vivo* following its over-expression in anaplastic thyroid carcinoma cells identify a protective role of D6/ACKR2 also in thyroid cancer. In fact, even so we were not able to demonstrate a slackening of tumor growth in nude mice injected with BHT D6 cells compared to that established in mice injected with BHT or BHT GFP cells, given that probably tumors have been excised too early (between 14th and 21th day after cell injection) to appreciate tumor volume differences, the absence of a dense macrophage infiltrate in BHT D6-derived tumors strongly suggests that their progression should be slower compared to that of highly infiltrated parental and control counterpart-derived tumors.

We have previously demonstrated that NF-κB is able to exert some of its pro-oncogenic activities in thyroid cancer through up-regulation of different target genes [27, 28] and of miR-146a [21]. Here we unveil a novel role of NF-κB in the regulation of the molecular mechanisms governing thyroid cancer. In thyroid neoplastic cells miR-146a up-regulation blocks D6/ACKR2 expression allowing chemokines to induce leukocytes migration in tumor microenvironment thereby promoting cancer progression. Thus, in thyroid carcinomas NF-κB indirectly blocks the expression of genes negatively regulating cancer growth.

The data presented in this paper highlight the strong association between cancer and inflammation in NF-κB-addicted thyroid neoplasms and point out tumor microenvironment as a reservoir of novel attractive molecular targets for advanced thyroid cancer treatment. Anaplastic thyroid tumors, in fact, even representing only 1% of thyroid carcinomas, still remain difficult to cure because of their aggressiveness and strong resistance to radio- and chemo-therapy [29]. Survival rate of ATC patients is about 4–12 months from the time of diagnosis, thus new therapeutical approaches beyond chemotherapy are needed. Restoration of D6/ACKR2 expression in ATC cells could be a novel strategy to block ATC progression by dampening inflammation in tumor microenvironment given the ability of D6/ACKR2 to scavenge chemokines so as to decrease leukocyte infiltration. Likely, since it is still complicated to obtain in clinical practice plasmid delivery to cancer cells for protein transgenic expression, re-expression of D6/ACKR2 could be performed by drug-induced inhibition of miR-146a. Of course, blocking miR-146a could have effects also on other molecular targets in ATC cells whose consequences we cannot foresee.

In conclusion, we believe that our findings add a novel tile in the puzzle of NF-κB-addicted thyroid cancer and that D6/ACKR2 and miR-146a are promising targets for new therapeutical strategies in fighting ATC.

## MATERIALS AND METHODS

### Cell culture and biological reagents

BHT101 cells, a kind gift of Prof. M. Santoro, Federico II University of Naples, were grown in Dulbecco's modified Eagle's medium (DMEM) (Sigma, St. Louis, MO USA) supplemented with 10% fetal bovine serum (Sigma). THP-1 cells were grown in RPMI 1640 medium (Sigma) supplemented with 10% fetal bovine serum (Sigma). To over-express D6/ACKR2 receptor, BHT101 cells were infected with pLenti-CMV-GFP-2A-Puro lentiviral vector (ABM, Richmond, BC, Canada) alone or containing the cDNA coding for human D6/ACKR2 fused in frame with FLAG tag. Anti-actin (sc-8432), anti-tubulin (sc-33749) and anti-IκBα (sc-371) were purchased from Santa Cruz Biotechnology, Inc. (Santa Cruz, CA), anti-FLAG (F7425 and F3165) were from Sigma, anti-D6/ACKR2 (human, PA5-11867) was from Pierce-Thermo Fisher Scientific (Waltham, MA USA), anti-F4/80 (MCA497R) was from AbD Serotec (Oxford, UK). Anti-PMCA (Plasma Membrane Calcium ATPase, clone 5F10) was from ABR Affinity BioReagents, Golden Co., USA. Anti-miR-146a was from Ambion-Thermo Fisher Scientific.

### Western blots

1 x 106 cells were incubated for 30 min at 4 °C in lysis buffer (20 mM Tris-HCl pH 7.5, 150 mM NaCl, 1 mM Na2EDTA, 1 mM EGTA, 1% Triton, 10% glycerol + protease inhibitors) and centrifuged at 14000 rpm for 10 min at 4 °C. Total proteins (20 µg) from supernatants were analyzed by 10% SDS-PAGE, under reducing conditions for PCMA, IκBα, tubulin and actin detection, and under non-reducing conditions for FLAG-D6/ACKR2 and endogenous D6/ACKR2 detection, and blotted onto nitrocellulose membrane (Schleicher & Schuell, Whatman GmbH). Filters were blocked for 90 min at room temperature with 5% non-fat dry milk in TBST buffer (10mM Tris-HCl (pH 8), 0.1% Tween 20, 150 mM NaCl) and incubated with 1:2000 dilution of anti-FLAG, anti-D6/ACKR2, anti-PCMA, anti-IκBα, anti-actin or anti-tubulin antibodies for 90 min. After TBST washing, blots were incubated for 1 h with horseradish peroxidase-conjugated secondary antibodies (GE Healthcare, UK) diluted 1:5000 in TBST buffer and then revealed by ECL (GE Healthcare, UK).

### Subcellular fractionation

1 x 107 cells were incubated in 1 ml of fractionation buffer (250 mM Sucrose, 20 mM HEPES pH 7.4, 10 mM KCl, 1.5 mM MgCl2, 1 mM EDTA, 1mM EGTA, 1 mM DTT and a protease inhibitors cocktail) and homogeneized in a Potter-Dounce. Then, after a 20 min. incubation on ice, lysates were centrifuged at 1000 g for 5 min at 4 °C to remove nuclei. Supernatants (cytosolic, mitochondrial and microsomal fraction) were centrifuged at 15000 g for 30 min at 4 °C to remove mitochondrial fraction. Supernatants from this last centrifugation wereultracentrifuged at 100000xg for 90 minutes at 4°C to pellet membrane vesicles (from endosomes, Golgi, plasma membrane, endoplasmic reticulum and secretory vesicles) contained in the microsomal fraction [22]. Microsomal pellets were resuspended in lysis buffer containing 1% SDS and used for Western blot analysis.

### Transwell migration assays

6 x 105 BHT, BHT GFP and BHT D6 cells were resuspended in 600 µl of Binding Buffer (serum-free Opti-MEM I, 4mM HEPES pH 7.4, 1% BSA) and incubated in the absence or in the presence of 1.2 nM CCL-2 or CCL-22 (R&D Systems, Minneapolis MN, USA) for 3 h at 37 °C. Additionally, 600 µl of Binding Buffer without cells were incubated in the absence or in the presence of 1.2 nM CCL-2 or CCL-2 for 3 h at 37 °C. After cell centrifugation, supernatants were collected. 1 x 106 THP-1 cells in 100 µl of serum-free Opti-MEM I (Invitrogen-Thermo Fisher Scientific, Waltham MA, USA) were placed on the polycarbonate membranes (5-mm pore size) on the bottoms of the upper compartment of the transwells (6.5 mm, Corning Life Sciences, Tewksbury MA, USA), while 600 µl of collected supernatants were added to the lower compartment. The plates were incubated at 37 °C in a 5% CO2 atmosphere saturated with H2O for 1 h. At the end of incubation, the cells at the upper side of the polycarbonate filter were mechanically removed. Cells that had migrated to the lower compartment through the filter were counted with a Neubauer chamber. The error bars represent technical replicates within a single experiment. Each experiment has been repeated at least three times.

### RNA extraction and mRNA quantification by real-time RT–PCR

Total RNAs from human normal (NT), papillary (P1, P2, P3, P7, P8, P9, P10) and anaplastic (A1, A3, A4, A16) thyroid tissues were kindly provided by Prof. Massimo Santoro, Federico II University of Naples. High quality total RNA preparations from cell lines and miR-146a detection by quantitative RT-PCR were carried out as previously described (19). To avoid the risk of DNA contamination in PCR assays, all RNA samples were treated with RNase free-DNase I (Promega, Fitchburg WI, USA) according to manufacturer's protocol. To analyze D6/ACKR2 mRNA expression, real-time reverse transcription–PCR was carried out with complementary DNAs reverse-transcribed from total RNA by using Transcription First Strand cDNA Synthesis kit and LightCycler 480 Probe Master Mix (Roche, Indianapolis, IN, USA), according to manufacturers’ procedure. Quantitative analysis was performed by LightCycler480 software (Roche) on the basis of the following protocol: denaturation step at 95 °C 10 min for 1 cycle, amplification steps at 95 °C 10 sec, 60 °C 10 sec, 72 °C 8 sec for 40 cycles. The error bars represent technical replicates within a single experiment. Each experiment has been repeated at least three times. Data were calculated by ΔCt method (2-ΔCt). The primers used were: human GAPDH, forward 5’-ATGGTGAAGGTCGGTGTGAAC-3’, reverse 5’-CCATGTAGTTGAGGTCAATGAAG-3’. Human D6/ACKR2, forward 5’-CTTCTCCAGTCACCGCTTC-3’, reverse 5’- AGTCATTTCCTCTTGGGCAG-3’.

### Luciferase assays

Validation of D6/ACKR2 as a target of miR-146a activity was performed by luciferase assays with pMIR-REPORT vector (Ambion-Thermo Fisher Scientific). The 3'UTR of D6/ACKR2 mRNA containing two adjacent putative sites (between nucleotides 111-125) of miR-146a binding was cloned downstream the cytomegalovirus-driven luciferase reporter gene in the pMIR-REPORT vector. The resulting plasmid (pMIR-D6 3'UTR) was used as a template for PCR generation of the mutant pMIR-D6 3'UTR Mut plasmid by the Quickchange II Site-Directed Mutagenesis kit (Agilent Technologies, Santa Clara CA, USA). Both plasmids were independently transfected in parental as well in scrambled and anti-miR-146a BHT101 cell lines to analyze the ability of D6/ACKR2 3'UTR to down-regulate luciferase activity in the presence or in the absence of miR-146a. Cells (4 x 105/well) were seeded in 6-well plate and, after 18h, were transfected with 1.5 µg of the pMIR-D6-Luc plasmid. Cell extracts were prepared 24h after transfection and reporter gene activity was determined by luminometer analysis (GloMax, Promega). A pMIR-REPORT-β-galactosidase vector (0.5 µg) was used to normalize for transfection efficiencies. The error bars represent technical replicates within a single experiment. Each experiment has been repeated at least three times.

### Tumor implantation and High Frequency Ultrasound

The animal protocols used in this work was evaluated and approved by the Animal Use and Ethic Committee (OPBA) of CEINGE, Biotecnologie Avanzate s.c.a.r.l. (Naples, Italy) (Protocol 27/2/14_n 2). It was in accordance with FELASA guidelines and the guidelines defined by the European Communities Council directive (2010/63/EU).

Twenty CD1, six-weeks-old, female mice (Charles River SRL, Italy) were anesthetized with isoflurane plus oxygen (induction phase: isoflurane 5% in oxygen 0.8 L/min; maintenance phase: isoflurane 1.5% in oxygen 0.8 L/min). High Frequency Ultrasound (HFUS) equipment, Vevo 2100 (VisualSonics Inc., Toronto, Ontario, Canada) mounting a 40 MHz probe was used in all procedures. Mice were injected with tumor cells subcutaneously on the right flank according to the following scheme: 6 mice were injected with 5x106 BHT cells in 200 µL, 6 mice were injected with 200 µL of 5x106 BHT GFP cells, 6 mice were injected with 5x106 BHT D6 cells, 2 mice were used as controls and were injected with 200 µL saline solution. Each mouse was positioned in dorsal recumbency on the handling table of the imaging station and a thick pad of warm gel was used over the tumor. After 14 days post tumor cells injection, mice were monitored once a week with HFUS to detect changes in tumor growth. For each tumor, mediolateral, anteroposterior and craniocaudal diameters were measured. Tumor volumes (TV) were calculated according to the formula V = (height x width x length x 3.16/6).

### Immunohistochemistry of tumor xenografts

Tumor xenografts were isolated, rinsed with PBS, fixed in 4% buffered neutral formalin for 24h at room temperature and embedded in paraffin after histological processing. Then, 5-6 µm thick paraffin sections were cut by microtome, deparaffinized and placed in a solution of absolute methanol and 0.3% hydrogen peroxide for 30 min to block endogenous peroxidase. Glasses were washed in PBS before immunostaining using Coplin jars and then incubated overnight at 4°C in a humidified chamber with rat anti-mouse F4/80 diluted 1:100 in PBS and subsequently incubated, first with biotinylated rabbit anti-rat IgG for 20 min (DAKO, Carpinteria, CA, USA), and then with streptavidin-HRP (DAKO) for 20 min. The development of signal was performed by incubating slides in diaminobenzidine (DAB-DAKO) solution containing 0.06 mM DAB and 2 mM hydrogen peroxide in 0.05% PBS pH 7.6 for 5 min, blocking the reaction in distilled water. Slides were washed, dehydrated with alcohol and xylene, and mounted with coverslips using a permanent mounting medium (Permount). F4/80 stained cells were scored by ImageJ software (NIH, Bethesda MD, USA). Results are ±SD of the number of positive F4/80 cells from six mice xenografts for each experimental group.

### Statistics

Data were analyzed with ANOVA and a Student's t test analysis. Data are presented as the means ±SD. P values <0.05 were considered significant.

## Abbreviations

NF-κB: nuclear factor-kappaB
IκBα: nuclear factor of kappa light polypeptide gene enhancer in B-cells inhibitor, alpha
UTR: untraslated region
SDS-PAGE: sodium dodecyl sulphate–polyacrylamide gel electrophoresis
RT-PCR: Real Time-Polymerase Chain Reaction
CCL-2: chemokine ligand-2
CCL-22: chemokine ligand-22
GFP: green fluorescence protein
